# Exploring changes in temporary abstinence in increasing and higher risk drinkers in England and Dry January participation in users of the Try Dry app in the UK between 2020 and 2021

**DOI:** 10.1186/s12889-022-14188-4

**Published:** 2022-09-26

**Authors:** Melissa Oldham, Inge Kersbergen, Sharon Cox, Jamie Brown, Richard Piper, Claire Garnett

**Affiliations:** 1 Department of Behavioural Science and Health, University College London, 1-19 Torrington Place, London, WC1E 7HB UK; 2 School of Health and Related Research, University of Sheffield, Sheffield, UK; 3 Department of Behavioural Science and Health, University College London, SPECTRUM Research Consortium, LondonEdinburgh, UK; 4 Alcohol Change UK, London, UK

**Keywords:** Alcohol, Dry January, Alcohol Reduction, Temporary Abstinence, Covid-19

## Abstract

**Background:**

We looked at changes in the prevalence of increasing and higher risk drinkers reporting a reduction attempt motivated by temporary abstinence and changes in prevalence of use of the official app accompanying Dry January between 2020 vs 2021, following the onset of the COVID-19 pandemic. We also explored potential shifts in the sociodemographic composition of both groups.

**Methods:**

We analysed data from: i) 1863 increasing and higher risk drinkers (defined as ≥ 8 on the AUDIT) responding to a nationally representative survey of adults in England in January and February 2020 and 2021, and ii) 104,598 users of the ‘Try Dry’ app, the official aid to those participating in Dry January 2020 and 2021 in the UK. We used logistic regression to examine shifts in the prevalence of increasing and higher risk drinkers reporting a reduction attempt motivated by temporary abstinence and explored whether there were shifts in the characteristics of this group in terms of AUDIT score, number of last year reduction attempts, smoking status, living alone, living with children, reducing alcohol consumption due to future health motives, age, sex, and occupational social grade between 2020 and 2021. We used t-tests and chi-squared tests to compare the prevalence of users of the ‘Try Dry’ app in 2020 and 2021 and examine whether the two groups differed in terms of age and sex.

**Results:**

The proportion of increasing and higher risk drinkers reporting a reduction attempt motivated by temporary abstinence increased from 4% in 2020 to 8% in 2021 (OR = 2.07, 95% CI = 1.38–3.11, *p* < .001) with no changes detected in sociodemographic composition. The number of *Try Dry* app users in 2021 increased by 34.8% relative to 2020. App users in 2021 were two years older on average [*p* < .001, d = .02], with a 2% increase in the proportion of female app users [*p* < .001, vs. < .01].

**Conclusions:**

Higher participation in Dry January 2021 relative to 2020 indicates increased engagement with a period of temporary abstinence following the COVID-19 related lockdowns in England and the UK, which is positive in the wider context of increasing alcohol consumption throughout the pandemic.

**Supplementary Information:**

The online version contains supplementary material available at 10.1186/s12889-022-14188-4.

## Background

Alcohol consumption is a dose-dependent [1], leading risk factor for preventable cases of cancer [2], linked with many other chronic and acute conditions [3]. Restrictions introduced as a result of COVID-19 in the UK have impacted on drinking behaviour with rises in increasing and higher risk drinking (defined by standard cut-offs of ≥ 8 on the full Alcohol Use Disorders Identification Test [4]) [6–8] and heavy episodic drinking [9]. As such, reducing alcohol consumption and associated harms is a public health priority [10]. This study reports changes in engagement with a national alcohol reduction campaign, ‘Dry January’ (a registered trademark), following the onset of the COVID-19 pandemic in the UK. Dry January aims to help people reduce their alcohol consumption. Here, we explore the level of participation in Dry January between 2020 and 2021, and potential shifts in the sociodemographic and drinking characteristics of those taking part. 

Dry January is a behaviour change programme created and run by the UK charity Alcohol Change UK (ACUK) that aims to help people to abstain from alcohol for the month of January and change habitual drinking patterns over the longer term [11]. Participants in Dry January are encouraged to use an app, ‘Try Dry’ (a registered trademark) to track alcohol units, calories and money saved, to track progress and to set future goals. Participants can also access daily coaching emails and peer support. Studies evaluating the impact of Dry January participation have found evidence of reported benefits to physical health [12, 13], mental health and well-being [12], as well as significant reductions in drinking days per week, drinks per typical drinking day and frequency of drunkenness six months after January [14]. Amongst participants of FebFast, a similar month-long abstinence challenge in Australia, 51% reported drinking less frequently four months after participation [15].

Lockdowns were first introduced in many countries in response to the COVID-19 pandemic, including England, Scotland and Wales in March 2020. In the UK, all non-essential stores and licenced premises were closed making social gathering and the opportunity to drink alcohol outside the home limited. While pubs, clubs and bars were closed, people could still purchase alcohol for home consumption and home alcohol expenditure increased in the first lockdown [16]. Evidence suggests that the first lockdown had a polarising impact on drinking patterns [8, 9] with 26% of drinkers drinking less and 26% drinking more [8]. The prevalence of increasing and higher risk drinking increased significantly with 1.8 times greater odds of increasing and higher risk drinking during lockdown relative to pre-lockdown [7]. As well as leading to increased alcohol consumption, the first lockdown also led to an increase in self-reported alcohol reduction attempts by increasing and higher risk drinkers (28.5% during lockdown vs. 15.3% pre-lockdown) [7].

A third lockdown, announced on the 4^th^ January and commencing on the 6^th^ January, coincided with the first week of Dry January in 2021. The impact of a third lockdown on alcohol consumption and reduction attempts may not have been the same as the first lockdown. A number of health behaviours, including alcohol consumption, changed over the course of the pandemic and in response to changing restrictions [17]. Deteriorating socio-economic, personal circumstances and mental health [18–20], could have led to substantial disengagement from the Dry January campaign. On the other hand, the pandemic could have refocussed attention on longer-term health and encouraged engagement with the campaign.

Beyond changes in the prevalence of participation in Dry January, the sociodemographic and drinking characteristics of participants may have changed. In previous years, being female and of more advantaged socio-economic position was associated with greater likelihood of participating in Dry January [21]. However, sociodemographic groups have been differentially affected by lockdown [8, 9, 17] and this has been reflected in drinking behaviour. Being younger [8], female [6, 8] and of a lower socio-economic position [6] were all associated with increased drinking in lockdown. Deterioration in psychological wellbeing and being a parent was associated with increases in the frequency of heavy episodic drinking [9]. Those groups drinking more in lockdown may be more likely to participate in Dry January if they are consciously aware of their increased drinking and are motivated to cut down. Conversely, the reasons leading to increased consumption may make these drinkers less likely to engage with a month of abstention. Understanding more about how the composition of Dry January participants changed during lockdown, may inform health communications and policy decisions around provision and targeting of support for alcohol reduction.

In this paper, we use data from two sources, i) the Alcohol Toolkit Study (ATS), a representative population survey in England, and ii) the database behind the Try Dry app, a freely available app built and run by ACUK. We compare the proportion of individuals who report a reduction attempt motivated by temporary abstinence in the ATS in England in January and February 2020 and 2021. We also examine whether there were shifts in and sociodemographic and drinking characteristics of increasing and higher risk drinkers making a reduction attempt motivated by temporary abstinence. We also compare the prevalence of Try Dry app use in the United Kingdom and compare the demographic characteristics of users, in 2021 relative to 2020. We aim to address the following research questions:

Among increasing and higher risk drinkers in England, has there been a change in the prevalence of reduction attempts motivated by temporary abstinence (e.g. Dry January) in 2021 compared with 2020?

Are drinking and sociodemographic characteristics associated with reduction attempts motivated by temporary abstinence (e.g. Dry January) amongst increasing and higher risk drinkers in England, and are any associations moderated by year?

Has there been a change in the number of users of the Try Dry app in the UK in January 2021 compared with January 2020?

Were there changes in the drinking and sociodemographic characteristics of users of the Try Dry app in the UK in January 2021 relative to January 2020?

## Methods

This protocol and analysis plan were pre-registered on the Open Science Framework (https://osf.io/dqukn/).

### Changes to pre-registered protocol

#### Comparison of temporary abstinence-motivated alcohol reduction attempts in 2021 compared with 2020 (RQ1 and 2)

In a deviation from our protocol, we report Ns (supplementary Table 1 and 2) rather than regression models and note that our sample size is insufficient to draw meaningful statistical inferences about region or ethnicity [22]. This is because there were very small sample sizes for those reporting reduction attempts motivated by temporary abstinence in some regions (as small as *n* = 1, see Supplementary Table 1) and amongst participants of minority ethnic groups (*n* = 0, see Supplementary Table 2).

#### Comparison of users of the Try Dry app in 2021 compared with 2020 (RQ3 and 4)

Incomplete location data for some participants in January 2020 meant that we were unable to isolate England only data and instead have used data from those in the UK. Finally, we did not examine differences in baseline AUDIT-C scores. The AUDIT-C was measured at multiple timepoints and as some participants had had the app for many years (e.g. reactivators) the data we used did not have the baseline AUDIT-C scores for the year of interest.

### Study design

#### Alcohol toolkit study (ATS)

The ATS is a monthly cross-sectional survey of a nationally representative sample of adults in England [23]. The study started in March 2014 and uses a form of random location sampling to select a new sample of approximately 1,700 adults each month (further details on the design and sampling methods of the ATS are described elsewhere [23]). Before COVID-19, surveys were conducted via face-to-face interviews but, due to social distancing restrictions, from April 2020 onward surveys were conducted by telephone. The telephone-based data collection relied on the same sampling and weighting approach as the face-to-face interviews, and diagnostic analyses indicate that comparisons between face-to-face and telephone data are reasonable [7].

For the present study, we used data from respondents to the survey aged 18 + in January and February 2020 and January and February 2021 who reported increasing and higher risk drinking. Increasing and higher risk drinking was defined by standard cut-offs of those scoring ≥ 8 on the full 10-item Alcohol Use Disorders Identification Test (AUDIT) or ≥ 5 on the AUDIT-C (questions 1–3 of the AUDIT) [4]. The outcome variable was based on a question about ‘recent cut down attempts’ and as such participants reporting a reduction attempt motivated by temporary abstinence in February were included in the analysis.

#### Try Dry app

The Try Dry app is the official digital aid to Dry January and is freely available to download. Users are given the option to enter their age and gender when they undertake the AUDIT-C within the app and are encouraged to log a status each day throughout January to specify whether they drank or not. In our protocol we specified that data for the ‘Try Dry’ analysis would be from those ‘who downloaded or reactivated the app in January 2020 or January 2021.’ However, this was loosely defined as many who downloaded the app for January 2021 may have done so in advance of January. For example, many download the app in late December in anticipation of participation. Rather than set an arbitrarily defined cut-off date for downloads, we define Try Dry app users as those who entered at least one status (e.g. logging a day as being dry or not) in January 2020 or 2021. This included users who downloaded the app for the first time, reactivators who had previously used the app, and continuous users who have been using the app throughout the year and not stopped using it in January.

### Measures

#### Comparison of temporary abstinence-motivated alcohol reduction attempts in 2021 compared with 2020 (RQ1 and 2)

The measures listed in this section apply to increasing and higher risk drinkers (defined as ≥ 8 on the AUDIT) from the ATS data. The primary dependent variable was a reduction attempt motivated by temporary abstinence. Participants who reported that they were making a current attempt to reduce their alcohol consumption or had made a serious attempt to reduce their alcohol consumption in the last year were asked a follow-up question about the motives underlying the most recent alcohol reduction attempt. They selected ‘yes’ or ‘no’ to a number of possible reasons for their most recent attempt to cut down including ‘to give up alcohol for a month (e.g. Dry January)’. Those who reported ‘yes’ in response to ‘to give up alcohol for a month (e.g. Dry January)’ were coded as 1, those who record ‘no’ were coded as 0. Increasing and higher risk drinkers not making any attempts to cut down on drinking in the last year were also coded as 0.

The following secondary outcomes were also explored in terms of how they varied by year (2020 vs. 2021); AUDIT score, number of last year reduction attempts, smoking status (never smoker [ref] vs. current vs. ex-smoker), living alone (vs. not), living with children (vs. not) and reducing alcohol consumption due to future health motives (vs. not). The following sociodemographic factors were also examined; age (as a continuous variable), sex (male [0]/female [1]) and occupational social grade in England (ABC1 high social grade vs. C2DE low social grade). See Supplementary Materials for more detail on the operationalisation and coding of measures.

#### Comparison of users of the Try Dry app in 2021 compared with 2020 (RQ3 and 4)

The primary dependent variable was the number of users of the Try Dry app in January 2020 and in January 2021.

Secondary outcomes were age and gender. Age was treated as a continuous variable. Gender was coded as men = 0, women = 1. App users reporting ‘other’ or ‘rather not say’ in response to their gender were not included in this analysis. These categories likely encompass within group variation in gender identity although this cannot be differentiated, and it was not meaningful to treat them as one analytic sample.

### Analysis

#### Comparison of temporary abstinence-motivated alcohol reduction attempts in 2021 compared with 2020 (RQ1 and 2)

Data from the ATS were weighted to match the English population profile on age, social grade, region, tenure, ethnicity, and working status within sex. Analyses focused on complete cases. The results section includes information on missing data.

Descriptive statistics were used to report the sociodemographic and baseline drinking characteristics of the sample by year. Descriptive statistics and logistic regression models were used to estimate the prevalence and odds of reduction attempts motivated by temporary abstinence by increasing and higher risk drinkers in the year 2020 (reference) vs. 2021 (RQ1). Due to the relatively small sample size (*n =* 1863), a series of logistic regression models, were conducted to examine changes in the drinking characteristics and sociodemographic composition of those reporting a reduction attempt motivated by temporary abstinence (RQ2). Each separate model contained year, characteristic and year by characteristic interaction terms.

#### Comparison of users of the Try Dry app in 2021 compared with 2020 (RQ3 and 4)

Descriptive statistics, t-tests and chi-squared tests were used to compare the number of users of the Try Dry app (RQ3) and the characteristics of users (RQ4; percentage of female users and mean age) in 2020 with 2021.

## Results

### ATS Sample characteristics

Six thousand seven hundred fifty nine people in England responded to the ATS during the study period (3,402 in January and February 2020, 3,357 responded in 2021). Of these respondents, 10.3% (*n* = 695) increasing and higher risk drinkers in England in January and February 2020 and 2021 reported a current or recent reduction attempt and indicated whether the reported reduction attempt was motivated by completing a period of temporary abstinence. A further 17.3% (*n* = 1,168) reported no attempts at cutting down in the last 12 months and were therefore coded as having no reduction attempts motivated by temporary abstinence and included in the final analytic sample of 1,863 (weighted *n* = 1,845). As the analytic sample is weighted throughout, the weighted sample characteristics are shown in Table 1.

**Table 1:** Weighted sample characteristics of increasing and higher risk drinkers in England, stratified by year

	Total	2020	2021	Missing^a^
Weighted n	1845	809	1036	-
Age, mean (SD)	45.74 (17.16)	44.27 (17.92)	46.89 (16.45)	14
Sex, % Female (n)	37% (689)	36% (288)	39% (401)	2^b^
Ethnicity, % White (n)	93% (1716)	95% (764)	92% (952)	6
Social grade, % ABC1 (n)	63% (1153)	69% (557)	59% (595)	23
AUDIT score, mean (SD)	8.43 (3.84)	8.22 (3.77)	8.59 (3.89)	30
Last year reduction attempts, mean (SD)	.92 (4.20)	.56 (2.60)	1.21 (5.11)	86
Currently trying to cut down	26% (475)	22% (180)	28% (295)	0
One or more attempt to cut down in past 12 months	33% (612)	27% (220)	37% (391)	0

^a^Missing data is from participants not responding to all questions

^b^Two participants responding ‘other’ to sex are excluded as the small samples prohibit meaningful comparison

### Proportion of increasing and higher risk drinkers reporting a temporary abstinence-motivated alcohol reduction attempt in 2020 versus 2021

The proportion of increasing and higher risk drinkers reporting a reduction attempt motivated by temporary abstinence increased significantly from 4% in 2020 to 8% in 2021 (OR = 2.07, 95%CI = 1.38, 3.11, *p* < 0.001).

### Sociodemographic and drinking correlates of temporary abstinence-motivated alcohol reduction attempts amongst increasing and higher risk drinkers 2020 versus 2021

People who attempted to reduce their consumption in the last year had greater odds of reporting a reduction attempt motivated by temporary abstinence (Table 2). No other significant associations or interactions were detected between the variables measured and a reduction attempt motivated by temporary abstinence. This suggests that there were no significant differences in the measured sociodemographic composition and drinking characteristics of increasing and higher risk drinkers attempting a Dry January in 2020 and 2021.

‘Try Dry’ sample characteristics.

The majority of ‘Try Dry’ app users (n = 104,598) in both years were female (68% in 2020 vs 70% in 2021). The average age of users was 40.5 (sd = 11.31) in 2020 and 42.8 (sd = 11.06) in 2021.

**Table 2:** Logistic regression models predicting temporary abstinence-motivated alcohol reduction attempts by increasing and higher risk drinkers by individual characteristics and characteristic by year interaction terms

		Individual characteristic				Characteristic*year interaction			
	Weighted n^a^	OR	95% CI		*P* value	OR	95% CI		*P* value
AUDIT score	1826	1.06	.92	1.23	.435	1.00	.92	1.08	.937
Last year reduction attempts	1764	1.18	1.02	1.36	.026	.93	.87	1.01	.079
Smoking status (never smokers [ref])									
Current smoker	1846	.57	.11	3.01	.509	1.35	.53	3.44	.531
Ex-smoker	1846	.74	.09	6.03	.781	.98	.30	3.23	.972
Living alone	1849	.60	.07	5.26	.644	1.12	.34	3.74	.856
Living with children	1856	.53	.11	2.67	.442	1.60	.63	4.03	.322
Future health Motive	1856	4.68	.70	31.35	.112	.82	.29	2.32	.710
Age	1841	1.02	.98	1.07	.259	.98	.96	1.01	.115
Sex^b^	1854	1.76	.41	7.53	.448	.80	.35	1.82	.594
Social grade^c^	1825	.78	.15	3.94	.764	1.60	.64	3.99	.317

^a^Due to sample size, individual regression models were run for each variable, N’s differ due to missing data

^b^Female as reference

^c^Higher social grade as reference

### Number of users of the Try Dry app 2020 versus 2021

There was a 38.4% increase in the number of Try Dry app users between 2020 and 2021 (see Table 3).

**Table 3:** Difference in sample characteristics of Try Dry app users in 2020 and 2021

	2020	2021	Difference
Try Dry app, n	43,868	60,730	
Gender, % Female (n)	68% (29,546^a^)	70% (33,390^b^)	x^2^ (df = 1) = 48.27, *p *< .001, V < .01
Age, mean (SD)	40.5 (11.31)^c^	42.8 (11.06)^d^	t (df = 91,889.99) = -31.03, *p* < .001, d = .20

^a^Missing gender data from 29 app users in 2020. 74 participants reporting ‘other’ and 499 reporting ‘rather not say’ are also treated as missing here

^b^Missing gender data from 10,237 app users in 2021. 58 reporting ‘other’ and 3017 reporting ‘rather not say’ are also treated as missing here

^c^Missing age data from 4 app users in 2020

^d^Missing age data from 10,131 in 2021

### Sociodemographic differences in Try Dry app users in 2020 versus 2021

Try Dry app users were significantly older in 2021 with an average age of 42.8 (SD = 11.06) relative to an average age of 40.5 (SD = 11.31) in 2020, although this age difference was small (d = 0.21). There were significantly more female app users in 2021 (70%) relative to 2020 (68%), but again the effect size was small (V < 0.01).

## Discussion

Increasing and higher risk drinkers were twice as likely to report a reduction attempt motivated by temporary abstinence in 2021 relative to 2020 in England, and there was a 34.8% increase in the number of users of the Try Dry app in January 2021 relative to 2020 in the UK. When looking at the sociodemographic and drinking characteristics of those reporting a reduction attempt motivated by temporary abstinence in 2021 relative to 2020, there is little evidence that the composition of participants has changed markedly. There were no significant interactions between sociodemographic and drinking characteristics and year, suggesting that the composition of participants was comparable between years despite the substantial increased participation in temporary abstinence-motivated alcohol reduction attempts in 2021. There were some significant differences in terms of the demographic composition of Try Dry users, with a higher proportion of female users and a slightly older group in 2021 relative to 2020. However, the effect sizes were small, with a two-percentage point increase in the proportion of female users and a 2.27 increase in mean age in years. Therefore, these identified differences might not represent meaningful changes in the demographic composition of Try Dry users. Indeed, the increase in age could be partially driven by natural aging of previous users of the app who reactivated or continued their use in January 2021.

Increases in alcohol consumption following COVID-19 lockdowns in the UK [7–9] are likely to have a negative impact on public health. However, the current study suggests there may be some evidence of increased engagement with a population-level intervention of temporary abstinence, with engagement doubling amongst increasing and higher risk drinkers. This is in line with previous literature outlining increased motivation to cut down on drinking after the initial COVID-19 lockdown in the UK [7]. These findings suggest that there could be increased motivation amongst some increasing and higher risk drinkers to reduce drinking, and amongst both increasing and higher risk drinkers and the general population to engage in temporary abstinence. More support should be directed at encouraging those motivated to cut down to engage with evidence-based approaches to do so. Furthermore, messaging around cutting down on alcohol consumption in public health campaigns such as ACUK’s future Dry January campaigns could be explicitly linked to the increases in drinking seen over the pandemic 19 and addressing habitual drinking and its impact on health, something that has been done with smoking cessation [25].

Previous research has demonstrated that women and those of higher socio-economic grade are more likely to participate in Dry January [21]. This was a pattern we did not detect in the ATS analysis of high and increasing risk drinkers. These differences may suggest that there are socioeconomic differences between increasing and higher risk drinkers engaging in reduction attempts motivated by temporary abstinence and those who use the Try Dry app. There is little evidence in this study of substantial shifts in terms of the sociodemographic or drinking characteristics of those using the app between 2020 and 2021, though we did not look at the socio-economic status of app users. As research has shown that alcohol harms are concentrated amongst more disadvantaged drinkers [26], research identifying the impact of COVID-19 on motivation to reduce alcohol consumption amongst more disadvantaged drinkers would be of value.

A strength of this study is the use of two data sources, a nationally representative survey and the official Dry January app to examine changes in the prevalence and characteristics of people engaging in both the official and unofficial forms of Dry January in 2020 and 2021. This triangulation from two data sources adds robustness to the findings over using individual data sets in isolation [27]. However, this approach is not without limitations. We cannot draw any conclusions about why a greater proportion of drinkers reported reduction attempts motivated by temporary abstinence and used the Try Dry app in 2021 relative to 2020. Participation in Dry January has increased each year since its inception in 2013, so the increases we see here may not directly relate to COVID-19 lockdowns or social distancing policies. The latest data from ACUK’s Dry January programme show that there were fewer new sign-ups in 2022 compared to 2021 [28], though there was an increase in the number of returners. This does perhaps indicate that the huge growth from 2020 to 2021 was not all ‘organic’ growth but may well have been partly driven by the pandemic. Future trends will help to further understand this increase. Finally, it is likely that many people engage in DIY attempts to stay dry in January whereby they attempt to complete the challenge but do not engage with the Dry January programme of support or engage with the Try Dry app. The ATS analysis cannot differentiate between unsupported attempts at temporary abstinence versus those engaging with the Try Dry app. As such, we cannot draw any conclusions about any changes in the group attempting not to drink in January but who did not engage with the Try Dry app between 2020 and 2021 both in terms of size and composition.

There were further limitations of the study. Our sample size was insufficient to draw meaningful statistical inferences about region or ethnicity. This was particularly limiting with regards to race and ethnicity, as there is evidence that COVID-19 has disproportionately affected Black, Hispanic and Asian groups [29–31]. Furthermore, online surveys and digital interventions often fail to capture the changing behavioural trends of those experiencing severe health and social comorbidities (e.g., homelessness, severe mental illness) and including those with an Alcohol Use Disorder. Previous research has shown that lockdown represented a risk factor for relapse for those with Alcohol Use Disorders who were previously abstinent and increased consumption amongst those still drinking [32]. This highlights the need for a specific focus on these groups as tailored and comprehensive approaches will be required.

## Conclusion

The proportion of increasing and higher risk drinkers in England reporting reduction attempts motivated by temporary abstinence doubled between 2020 and 2021, alongside a 38.4% increase in the number of users of the Dry January app, Try Dry. There was limited evidence of changes in the sociodemographic composition of those participating in Dry January 2021 relative to 2020, with small increases in the average age of Try Dry app users and the proportion of female users. These findings may be indicative of increases in motivation to reduce drinking and to engage with a period of temporary abstinence following the COVID-19 related lockdowns in England and the UK. Increasing participation in Dry January and increases in the proportion of increasing and higher risk drinkers reporting reduction attempts motivated by temporary abstinence is encouraging within the context of increasing alcohol consumption throughout the pandemic.

## Supplementary information

**Additional file 1: Supplementary Table 1: **Number of participants from each region reporting a Dry January motivated alcohol reduction attempt in 2020 and 2021.** Supplementary Table 2: **Number of white and non-white participants reporting a Dry January motivated alcohol reduction attempt in 2020 and 2021.

## Data Availability

The datasets analysed during the current study are available from the corresponding author on reasonable request.
