# Supplementary material for: Exploring changes in temporary abstinence in increasing and higher risk drinkers in England and Dry January participation in users of the Try Dry app in the UK between 2020 and 2021
Source: BMC Public Health. 2022 Sep 26;22:1822. doi: 10.1186/s12889-022-14188-4 (PMC9510241; doi:10.1186/s12889-022-14188-4)
Supplement: Supplementary file 1 — Additional file 1: Supplementary Table 1: Number of participants from each region reporting a Dry January motivated alcohol reduction attempt in 2020 and 2021. Supplementary Table 2: Number of white and non-white participants reporting a Dry January motivated alcohol reduction attempt in 2020 and 2021. [file 12889_2022_14188_MOESM1_ESM.docx]

**Supplementary Materials**

**Measures**

**Comparison of Dry January-motivated alcohol reduction attempts in 2021 compared with 2020 (RQ1 and 2)**

**Dependent Variable**

Participation in Dry January

Participants who reported making a current attempt to reduce their alcohol consumption or making a serious attempt to reduce their alcohol consumption in the last year were asked a follow-up question about the motives underlying the most recent alcohol reduction attempt. Respondents were asked ‘Which of the following, if any, do you think contributed to you making the most recent attempt to cut down?’ and given a list of possible options. Those reporting ‘yes’ in response to ‘to give up alcohol for a month (e.g. Dry January)’ were coded as 1, those who record ‘no’ were coded as 0. Increasing and higher risk drinkers not making any attempts to cut down on drinking in the last year were also coded as 0.

**Predictor Variables**

Year

Whether the respondent answered in 2020 or 2021

AUDIT score

Assessed using the Alcohol Use Disorders Identification Test (AUDIT), a 10-item screening tool developed by the World Health Organisation, with a score ranging from 0 to 40. Our sample is defined as having a score of 8 or more.

Last year reduction attempts

Measured by the question “How many serious attempts to cut down on your drinking alcohol have you made in the last 12 months?”. The answer was entered as a continuous value.

Smoking Status

Smoking status was measured with the question ‘which statement about tobacco use and cigarette smoking best describes you?’, with the options i) ‘I smoke cigarettes (including hand-rolled) every day’, ii) ‘I smoke cigarettes (including hand-rolled), but not every day’, iii) ‘I do not smoke cigarettes at all, but I do smoke tobacco of some kind (e.g. pipe, cigar or shisha)’, iv) ‘I have stopped smoking completely in the last year’, v) ‘I stopped smoking completely more than a year ago’ and vi) ‘I have never smoked any cigarettes. Smoking status was derived with those choosing vi coded as a never smoker (reference group), those choosing i, ii or iii coded as a current smoker and those choosing iv or v coded as an ex smoker.

Living Alone

This was derived from the “number in household” measure with those reporting a household of one coded as 1, and those reporting more than one (i.e. not living alone) coded as 0.

Living with Children

This was derived from the “number of children” measure with those reporting between 1-9 children coded as 1, and those reporting no children coded as 0.

Future Health Motives

Measured by the question ‘Which of the following, if any, do you think contributed to you making the most recent attempt to cut down?’. This is the same question used to operationalise the dependent variable and participants could select yes or no to multiple answers. Those reporting ‘yes’ in response to ‘concern about future health problems’ were coded as 1, those who record ‘no’ were coded as 0. Those not making a reduction attempt were also coded as 0.

Sociodemographic Factors

Sociodemographic characteristics included age (as a continuous variable), sex (male [0]/female [1]), ethnicity and occupational social grade in England. Due to a representative sample with a high proportion of white respondents, ethnicity was categorised as White = 1, Minority Ethnic = 0. Occupational Social grade was categorised as ABC1 (which includes managerial, professional and intermediate occupations as the reference group) versus C2DE (which includes small employers and own‐account workers, lower supervisory and technical occupations and semi‐routine and routine occupations, never workers and long‐term unemployed).

**Comparison of users of the Try Dry app in 2021 compared with 2020 (RQ3 and 4)**

**Dependent Variable**

App Users

The number of users of the Try Dry app in January 2020 and in January 2021.

**Predictor Variables**

Sociodemographic Factors

Age was treated as a continuous variable. Sex was coded as men =0, women = 1. App users reporting ‘other’ or ‘rather not say’ in response to their gender were not included in this analysis. These categories likely encompass within group variation in gender identity and it is not meaningful to treat them as one analytic sample.

**Supplementary Table 1:** Number of participants from each region reporting a Dry January motivated alcohol reduction attempt in 2020 and 2021

|  | **Dry January motivated reduction** | | **No Dry January motivated reduction** | |
| --- | --- | --- | --- | --- |
|  | **2020** | **2021** | **2020** | **2021** |
| **North East** | 1 | 6 | 41 | 51 |
| **North West** | 1 | 12 | 112 | 149 |
| **Yorkshire and the Humber** | 5 | 15 | 103 | 89 |
| **East Midlands** | 3 | 6 | 65 | 74 |
| **West Midlands** | 2 | 7 | 100 | 108 |
| **East of England** | 4 | 8 | 57 | 101 |
| **London** | 14 | 19 | 117 | 112 |
| **South East** | 3 | 8 | 89 | 160 |
| **South West** | 1 | 6 | 91 | 106 |
| **Total** | 34 | 87 | 775 | 950 |

**Supplementary Table 2:** Number of white and non-white participants reporting a Dry January motivated alcohol reduction attempt in 2020 and 2021

|  | **Dry January motivated reduction** | | **No Dry January motivated reduction** | |
| --- | --- | --- | --- | --- |
|  | 2020 | 2021 | 2020 | 2021 |
| **White** | 34 | 80 | 730 | 872 |
| **Non White** | 0 | 6 | 44 | 74 |
| **Total** | 34 | 86 | 774 | 946 |
